# A FT-NIR Process Analytical Technology Approach for Milk Renneting Control

**DOI:** 10.3390/foods11010033

**Published:** 2021-12-23

**Authors:** Silvia Grassi, Lorenzo Strani, Cristina Alamprese, Nicolò Pricca, Ernestina Casiraghi, Giovanni Cabassi

**Affiliations:** 1 Department of Food, Environmental and Nutritional Sciences, Università degli Studi di Milano, Via Giovanni Celoria 2, 20133 Milan, Italy; silvia.grassi@unimi.it (S.G.); lorenzo.strani@unimi.it (L.S.); ernestina.casiraghi@unimi.it (E.C.); 2 Department of Chemical and Geological Sciences, University of Modena and Reggio Emilia, Via Campi 103, 41125 Modena, Italy; 3 Centro di ricerca Zootecnia e Acquacoltura (CREA-ZA), Consiglio per la Ricerca in Agricoltura e l’Analisi dell’Economia Agraria, Via Antonio Lombardo 11, 26900 Lodi, Italy; nicolo.pricca@crea.gov.it (N.P.); giovanni.cabassi@crea.gov.it (G.C.)

**Keywords:** FT-NIR spectroscopy, Industry 4.0, milk coagulation, multivariate statistical process control charts, MSPC, PAT, skimmed milk powder

## Abstract

The study proposes a process analytical technology (PAT) approach for the control of milk coagulation through near infrared spectroscopy (NIRS), computing multivariate statistical process control (MSPC) charts, based on principal component analysis (PCA). Reconstituted skimmed milk and commercial pasteurized skimmed milk were mixed at two different ratios (60:40 and 40:60). Each mix ratio was prepared in six replicates and used for coagulation trials, monitored by fundamental rheology, as a reference method, and NIRS by inserting a probe directly in the coagulation vat and collecting spectra at two different acquisition times, i.e., 60 s or 10 s. Furthermore, three failure coagulation trials were performed, deliberately changing temperature or rennet and CaCl_2_ concentration. The comparison with fundamental rheology results confirmed the effectiveness of NIRS to monitor milk renneting. The reduced spectral acquisition time (10 s) showed data highly correlated (r > 0.99) to those acquired with longer acquisition time. The developed decision trees, based on PC1 scores and T^2^ MSPC charts, confirmed the suitability of the proposed approach for the prediction of coagulation times and for the detection of possible failures. In conclusion, the work provides a robust but simple PAT approach to assist cheesemakers in monitoring the coagulation step in real-time.

## 1. Introduction

The dairy industry is moving towards the Industry 4.0 concept, which aims at digitalization and automation across the entire production chain by increasing connectivity from raw materials to final product [1]. Industry 4.0 calls for process analytical technology (PAT), which ensures final product quality through real-time measurements of performance attributes along the whole production process, thus reducing process cycle time, replacing costly laboratory tests, enhancing efficiency, minimising waste, and enabling continuous learning [2]. 

The key point of PAT is the use of analysers, i.e., measurement systems and sensors, suitable for implementation in the process line. Indeed, several criteria need to be considered prior to employing PAT tools in a process [3], as follows: (1) capability to monitor the desired performance attributes, (2) suitability of their implementation along the process streams to monitor the required information with adequate robustness, (3) fulfilment of the optimal measurement conditions to obtain reliable data, and (4) guaranteed maintenance and validation of their performance over time. 

The PAT approaches are particularly relevant when manufacturing protocols are modified. For instance, in cheese production the use of skimmed milk powder is still common for milk standardization in Countries where the availability of raw milk is scarce or subjected to periodic fluctuations in market price [4,5,6]. Even though in some Countries, such as Italy, the use of milk powder or reconstituted milk is still forbidden in cheese manufacturing [7], PAT implementation in case of reconstituted milk use would be beneficial for local dairy processing industries of many other Countries.

Panikuttira et al. [1] remarked how PAT tools implemented in the dairy chain moved from univariate measuring systems, determining pH, temperature, pressure, and flow, to high throughput measuring systems, such as spectroscopic methods. Among spectroscopic sensors, near infrared spectroscopy (NIRS) has been widely explored in the food sector, demonstrating the capability of handling the complexity and high variability of food and the dynamic nature of food processing [8]. However, NIR spectra are mainly characterised by broad bands, arising from overlapped overtone and combination bands, caused by the interaction of infrared radiation and molecular groups of the various components of food matrices. Therefore, powerful data analysis tools are needed to model relevant information retained in the NIR signal. Within the four levels of chemometric analysis in the PAT framework identified by Wold et al. [9], a pivotal step is the monitoring of the evolution of a batch process by means of multivariate measurements, using the process and raw material data to classify the process as acceptable or not.

By moving from a univariate measuring system to a multivariate one, monitoring of the quality should move from traditional statistical quality control (SQC) to multivariate statistical process control (MSPC). Indeed, SQC is meant to determine whether the variability of one parameter is under control by representing the measured value on a univariate control chart with appropriate limits. The univariate SQC chart implicates the risk of having the measured quality parameter within acceptable limits, even if the process is out of control due to other quality parameters and/or their interactions, outside of the specifications [10]. On the other hand, a multivariate measuring system, such as NIRS, provides for each sampling point hundreds of highly correlated variables that can be handled by multivariate projection methods, such as principal component analysis (PCA) and partial least squares (PLS), enabling the reduction in the data dimensionality by taking advantage of its correlated structure [11]. The principal components (PCs) or latent variables (LVs) needed for the description of the process variability could then be used for MSPC chart construction by representing these components against each other or against process time. However, when more than one PC or LV is required to describe the main variability of the process, the inspection of all their combinations can be useful. Thus, a different strategy could be more convenient to represent the overall variability of the process with a single chart based on Hotelling’s T^2^ [11]. In any case, the use of MSPC charts based on NIR signal would provide the visualization of the whole changes occurring along the process, overcoming the inspection of a single process variable.

Multivariate statistical process control (MSPC) charts have been used in the dairy industry for fault diagnosis in the monitoring of milk pasteurisation process [12]. However, limited research has been performed in the development of MSPC based on NIR signal for milk coagulation monitoring [13]. On the other hand, different chemometric strategies have been applied to monitor coagulation and syneresis kinetics [14,15,16,17,18], thus demonstrating the reliability of NIRS. Even though the work by Grassi et al. [13] extensively explored the physicochemical and process factors affecting both the NIR signal and the coagulation occurrence, the proposed approach was far from being suitable for industrial implementation. Indeed, the NIRS acquisition interval was high (60 s) compared with gelation time and the data analysis was too complex, being based on the development of a multivariate curve resolution model to extract useful data for MSPC chart construction.

The present work aims to close the gap between the scientific reliability of NIRS in coagulation monitoring and the industrial criteria needed for employing PAT tools in cheesemaking. Thus, the NIR spectra were compared with fundamental rheology analyses to assess the capability of NIRS to monitor the coagulation progress of commercial pasteurised skimmed milk combined with reconstituted low-heat, skimmed milk powder. Furthermore, a fast spectrum collection procedure was tested by reducing the scanning time from 60 s to 10 s to adapt the method to real-time coagulation monitoring. Finally, decision trees, based on MSPC charts, were developed as PAT tools for simple prediction of coagulation time and early failure detection in milk coagulation.

## 2. Materials and Methods

### 2.1. Sample Preparation and Coagulation Experiments

Low-heat, skimmed milk powder (EPI ingredients, Nantes, France) was dissolved in 300 mL distilled water (24 ± 1 °C) at 11.5 g/100 mL to yield reconstituted milk sample with a protein content of 3.4 ± 0.1 g/100 g. Complete powder dissolution was achieved by mixing with a magnetic stirrer bar at room temperature for 20 min. The reconstituted sample was mixed with commercial pasteurised skimmed milk (Granarolo, Bologna, Italy; protein content 3.2 g/100 mL) in 40:60 (EPI40) and 60:40 (EPI60) ratios, for a total of six replicates for each condition. 

The coagulation experiments were carried out by the addition of 1 mmol/L of CaCl_2_ (0.0035 g/L) by using a stock solution (50 g/L), and of rennet (Naturen^®^ 220 CHR Hansen, Hoersholm, Denmark) at a final concentration of 0.088 IMCU/g of milk. The coagulation temperature was set at 37 °C.

Furthermore, three failure batches (FB1:FB3) were set up, as follows: in FB1, milk heating was turned off after rennet addition; in FB2, half of the rennet amount was added; in FB3, half of the CaCl_2_ amount was added.

### 2.2. Fundamental Rheology

Milk renneting trials were monitored by performing a time–curing test in oscillation by means of a Physica MCR 102 rheometer (Anton Paar GmbH, Graz, Austria), controlled by the RheoCompass software (v. 1.21.652, Anton Paar, Graz, Austria). Each sample (19 mL) was poured in the concentric cylinders (CC27) of the rheometer and the test was performed for 40 min at 37.0 ± 0.1 °C, applying constant strain (0.01%) and frequency (1 Hz) values. Values of elastic (G′) and viscous (G″) moduli were registered continuously to measure changes in the viscoelastic behaviour of the samples during renneting.

### 2.3. FT-NIR Spectroscopy

Rennet coagulation of each sample, placed in a thermostatic bath at 37.0 ± 0.5 °C, was continuously monitored for 40 min with a Fourier transform (FT)-NIR spectrometer (MPA, Bruker Optics, Milan, Italy). Spectral acquisition was performed by means of a transflectance fibre optic probe (0.2 cm effective pathlength) directly inserted in the sample, in the 12,500–4000 cm^−1^ spectral range, with a resolution of 8 cm^−1^. A total of 3 coagulation replicates were analysed for each milk mixture by acquiring spectra every 60 s, as the result of 64 scans (64-scan procedure). The other 3 coagulation trials for each milk mixture were analysed in the same conditions, but with the number of scans reduced from 64 to 9 (9-scan procedure) to shorten the acquisition time from 60 to 10 s and increase the acquisition frequency (6 spectra/min vs. 1 spectrum/min). This procedure allows a timely intervention in case of failures.

OPUS software (v. 6.0, Bruker Optics, Milan, Italy) was used to manage the instrument.

### 2.4. Data Analysis

The FT-NIR spectra were reduced in the range 12,500–5824 cm^−1^ and pre-processed by standard normal variate (SNV). 

Spectra acquired by the 64-scan procedure were compared with the ones collected by the 9-scan procedure by Pearson correlation to assess if the two approaches provide comparable information.

PCA–MSPC charts were built by a PCA model calibrated with the FT-NIR spectra collected for the three coagulation replicates of EPI40 and EPI60 (EPI40-R1, EPI40-R2, EPI40-R3, EPI60-R1, EPI60-R2, and EPI60-R3) analysed by the 64-scan procedure (240 spectra × 1840 wavenumbers). The PCA model was then tested in prediction with the FT-NIR spectra collected with the 9-scan procedure applied to the replicated coagulations of EPI40 and EPI60 (EPI40-A, EPI40-B, EPI40-C, EPI60-A, EPI60-B, and EPI60-C), and the failure batches (FB-1, FB2, and FB3). Decision trees were constructed using delta PC1 scores and delta normalised Hotelling’s T^2^ statistics to provide an automated decision tree procedure for real-time coagulation control. 

Spectral data were processed and analysed with self-constructed routines and toolboxes in Matlab environment (the Mathworks Inc., Natick, MA, USA).

## 3. Results and Discussion

### 3.1. Capability to Monitor the Desired Quality and Performance Attributes

**Figure 1:**
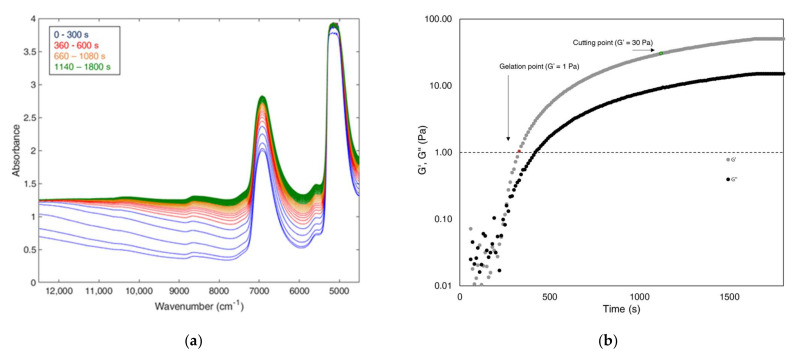
Line monitoring of the trial EPI60-R1: (**a**) FT-NIR spectra collected by the 64-scan procedure; (**b**) elastic (G′) and loss (G″) modulus curves obtained from the time–curing test.

The FT-NIR spectra collected by the 64-scan procedure showed a characteristic behaviour, already reported by Strani et al. [19]. The spectra collected at the beginning of the coagulation and the ones collected at the end presented similar bands (Figure 1a): a band at 6900 cm^−1^, ascribable to symmetric and asymmetric stretching of O-H water bonds, and two bands at 10,800 and 8600 cm^−1^, linked to C-H bonds [20,21]. The main changes occurring during coagulation are due to the scattering effect linked to changes in particle size [22], i.e., the increased dimensions of casein micelles, which can strongly vary in diameter (80–300 nm) [23] and number [24]. This modification occurred in the first minutes of coagulation. Indeed, in the trial EPI60-R1, the first five spectra (represented as blue spectra in Figure 1a), corresponding to the first five minutes of coagulation, are affected by a fast increase in the baseline offset; after that, it is possible to notice a change in the slope between 12,500–9000 cm^−1^ up to ten minutes of coagulation (represented by spectra coloured in red). From this point on, the differences are lower and only a slight increase in the slope is observable (orange and green spectra). The modification in baseline slope and 6900 cm^−1^ band are related to the overall water absorption due to the changed physical properties of the medium [25].

To confirm that the observed changes are linked to the curd development, the rheological behaviour of the coagulating milk was monitored, measuring the elastic modulus (G′) and the loss modulus (G″) over the coagulation time [26]; Figure 1b reports an example of the evolution of the two moduli for EPI60-R1. The G′ value defines the degree of solid-like character of the gel, whereas the G″ value indicates the degree of the liquid-like behaviour. Thus, when the gel begins to form, G′ and G″ values rapidly increase, with a higher rate for G′. Referring to Arango et al. [16], the rheological gelation time, corresponding to a G′ values of 1 Pa, and the rheological cutting time, corresponding to a G′ of 30 Pa, were extrapolated for all the mix ratio trials (Table 1). 

The rheological and FT-NIR spectral behaviours were compared. Considering the trial EPI60-R1, it was possible to notice that the rheological gelation point occurring after 330 s (red dot in Figure 1b) corresponded with the first red spectrum in Figure 1a, when the slowdown of the baseline drift was observed. After the rheological cutting point (green dot in Figure 1b), the spectra resulted constant in shape and slope (green spectra in Figure 1a).

### 3.2. Assessment of the Optimal FT-NIRS Measurement Conditions to Obtain Reliable Data

**Figure 2:**
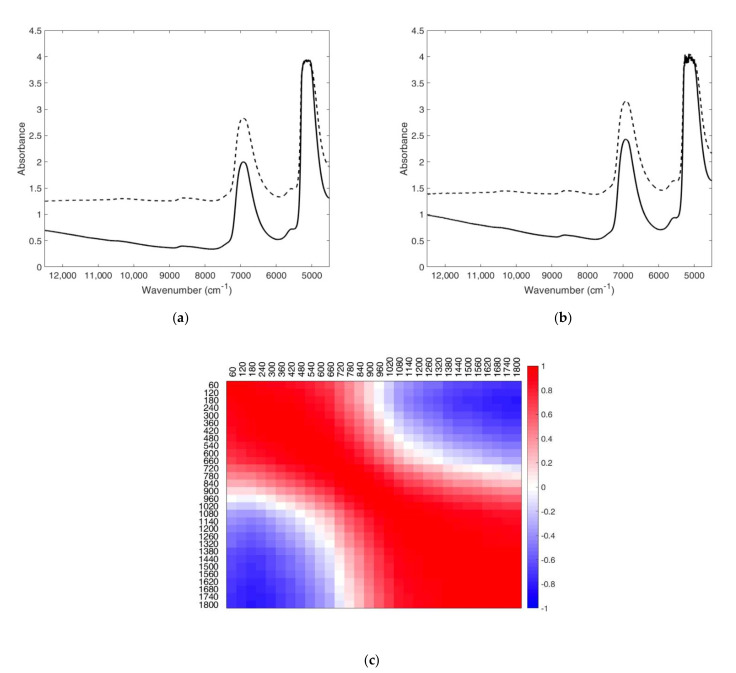
FT-NIR data for the trial EPI60-R1: (**a**) spectra collected at the beginning (bold line) and at the end of the coagulation (dashed line) by the 64-scan procedure; (**b**) spectra collected at the beginning (bold line) and at the end of the coagulation (dashed line) by the 9-scan procedure; (**c**) correlation coefficient map of the spectra collected from 60 to 1800 s of coagulation.

Even though the capability of FT-NIRS to monitor milk coagulation was demonstrated, the 64-scan procedure required too high an acquisition time (60 s). Therefore, a faster strategy was tested, decreasing the acquisition time to 10 s, by reducing the number of scans to 9, thus increasing the spectral acquisition frequency. The time reduction would allow the implementation of the proposed method to real-time coagulation monitoring. To assess the reliability of the 9-scan approach, each spectrum acquired by the 64-scan procedure was compared with the corresponding spectrum collected with the faster procedures (9 scans, 10 s). The spectra collected by both approaches showed the same modifications in the FT-NIR signal (Figure 2a,b).

Furthermore, the Pearson correlation returned coefficients (r) higher than 0.99 for all the spectra collected at the same time. An example of correlation coefficients obtained for the trial EPI60-R1 is reported in Figure 2c.

These results proved that a reduced scan number could be used for spectra acquisition without loss of information, thus guarantying the acquisition of a spectra every 10 s, a suitable timing for real-time coagulation process monitoring.

### 3.3. Suitability of FT-NIRS Implementation to Monitor Coagulation Progress

#### 3.3.1. Principal Component Analysis

**Figure 3:**
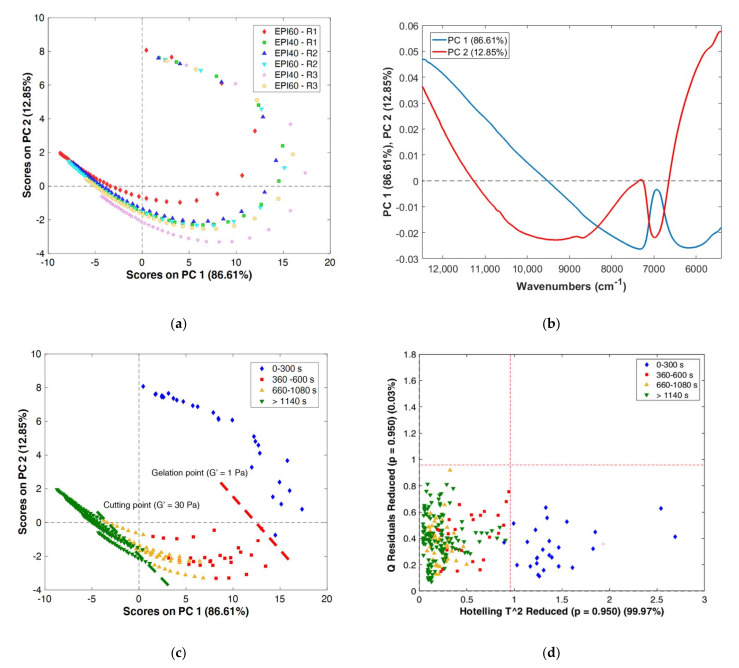
Principal component analysis of the calibration dataset: (**a**) score plot PC1 vs. PC2 with samples coloured according to the trial; (**b**) loading plot of PC1 and PC2; (**c**) score plot PC1 vs. PC2 with samples coloured according to coagulation time; (**d**) Q-residuals vs. T^2^ plot with samples coloured according to coagulation time. R1, R2 and R3 correspond to the three replicates of each mix ratio tested.

A PCA was performed on the calibration dataset constituted of the 120 FT–NIR spectra collected for the three technological replicates of EPI40 and EPI60 coagulation analysed with the 64-scan procedure. The first two components described 99.46% of the total variance of the system. The coagulation trend of all the trials followed a similar pattern (Figure 3a) mainly related to the decreasing of PC1 in the range 12,000–7500 cm^−1^, modelling the residual multiplicative effects of scattering after SNV treatment evolving during coagulation time, and the modification of the band at 6900 cm^−1^ related to O–H first overtone (Figure 3b), both linked to the change in the scattering properties of the medium. The curved behaviour of the scores was also caused by SNV pre-treatment (Figure 3a), as explained by Fearn et al. [27]; however, in this case, the pre-treatment appeared to be suitable to cope with scattering phenomena not related to the coagulation.

By colouring the samples according to different coagulation times (Figure 3c) it was possible to notice that spectra collected at the beginning of the process (between 0 and 300 s) had assumed positive PC1 and PC2 scores, representing the phase in which the milk is still liquid. Positive PC1 and PC2 values corresponded to spectral signals, characterised by a remarkable slope in the baseline between 12,500 and 9000 cm^−1^ and a lower absorption in the peak at 6900 cm^−1^ (Figure 3b). This phase of coagulation involves the k-casein proteolysis to para-casein. The spectra collected from 360 to 1080 s after the rennet addition were mainly positioned in the IV quadrant of the score plot, and reasonably corresponded to the phase of aggregation of para-casein leading to gel network formation. After 1140 s of monitoring, the samples were affected by smaller modifications, remarked by their lower dispersion in the score plot, confirming the formation of a continuous 3D protein network and corresponding to G′ values higher than 30 Pa. 

Figure 3d presents the Hotelling’s T^2^ vs. Q-statistic plot for model diagnostic. All the process variation was well represented by the PCA model; indeed, all the samples were located in the confidence limit of Q-statistic (*p* = 0.95). The samples collected before the rheological gelation time (in blue) had high Mahalanobis distance from the centre of the PCA model (Hotelling’s T^2^). The PCA model diagnostic suggested that T^2^ could be used to describe the coagulation occurrence as a measure of the transition toward a more regular and reproducible structure of the gel phase.

#### 3.3.2. Multivariate Statistical Process Control Charts

**Figure 4:**
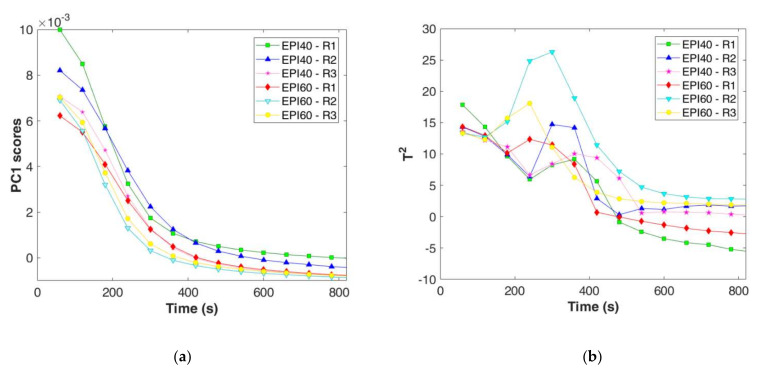
Multivariate statistical process control chart for the calibration dataset: (**a**) zoom on PC1–MSPC chart in the first 800 s; (**b**) zoom on T^2^–MSPC chart in the first 800 s.

Recognising the coagulation phases is quite important for process understanding, however when moving to process control it is necessary to identify quite early if the process is under control, in order to plan the needed actions. This could be solved by the construction of MSPC charts followed by a decision tree to identify the key action points. To the aim, PC1 scores were extracted from the PCA calibration phase and reported as function of the coagulation time (Figure 4a). PC1 scores of the spectra collected every 60 s showed a fast decrease with a remarkable slow down after 300 s and 360 s for EPI60 and EPI40, respectively. After that, scores reached a plateau after 500–600 s of process monitoring. 

Similarly, T^2^ values were extracted from the PCA calibration model (Figure 4b). Here, a first minimum was detected after 120–180 s (EPI60) and 240 s (EPI40), followed by a fast increase until a maximum at 300–360 s, according to the considered trial, and a decrease up to a plateau for all the trials after 600 s. It seems that the PC1 scores and T^2^ trends could be useful to describe both coagulation start and end in accordance with the rheological results (Table 1). However, to confirm the reliability of the results, data acquired at higher measurement frequency are discussed hereafter. 

**Figure 5:**
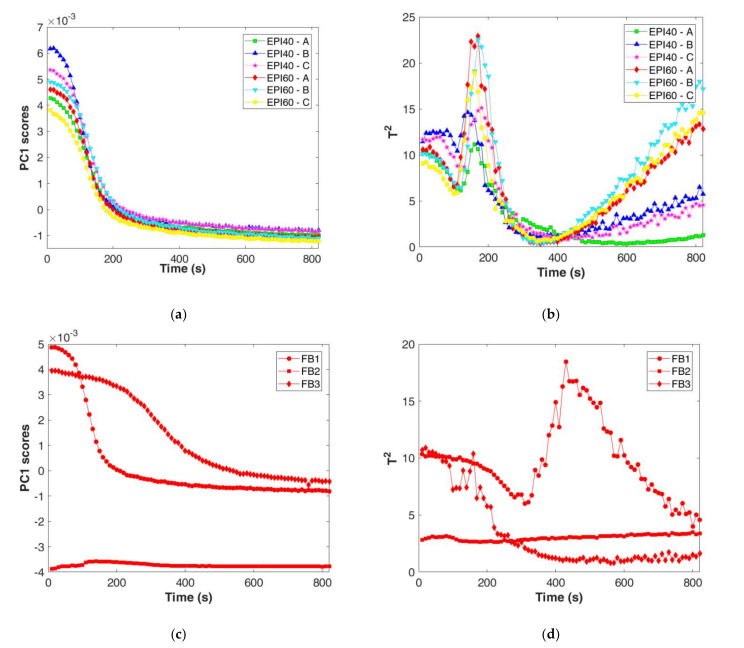
Multivariate statistical process control chart for the test datasets: (**a**) zoom on PC1–MSPC chart in the first 800 s for EPI40 and EPI60; (**b**) zoom on T^2^–MSPC chart in the first 800 s for EPI40 and EPI60. (**c**) PC1–MSPC chart for failure batches (FB1, FB2, and FB3); (**d**) T^2^–MSPC chart for failure batches (FB1, FB2, and FB3).

The spectra collected by the 9-scan procedure, for both the EPI40 and EPI60 replicates and the 3 failure batches, were projected on the space defined by the PCA model. PC1 scores and T^2^ of measurements recorded every 10 s (Figure 5a,b) confirmed the trend previously observed for the 64-scan procedure. The EPI60 replicates (A, B, and C) were characterised by a maximum decreasing rate of PC1 scores at 110–120 s, whereas for EPI40 it was delayed at around 130–150 s. The timely recording permitted to notice that the EPI40 delay was not as long as the one modelled by the 64-scan acquisition procedure. The T^2^ values decreased in the first seconds of the process up to 100–110 s and 110–120 s for EPI60 and EPI40, respectively (Figure 5b). Then, the T^2^ values increased rapidly up to a peak value, between 140 and 180 s according to the considered trial, and just after the peak value they quickly decreased. After 400 s, EPI60 replicates increase again in Hotelling’s T^2^ denoting a higher Mahalanobis distance from the centre of the PCA model. However, the study of this change is out of the scope of the present study and should be better investigated in the future.

The projection of failure batches demonstrated that the model discriminated off-specific batches from both PC1 and T^2^ control charts. The failure in temperature control (FB1) led to a delay in the PC1 slow down, whereas the reduction in rennet addition (FB2) resulted in negative PC1 scores with small variation over time (Figure 5c). For all the failure batches tested, the T^2^ control chart confirmed the out of compliance through the delay (FB1) or the absence of the peak values (FB2 and FB3); furthermore, FB3 showed an irregular behaviour after the first minutes of monitoring (Figure 5d). 

**Figure 6:**
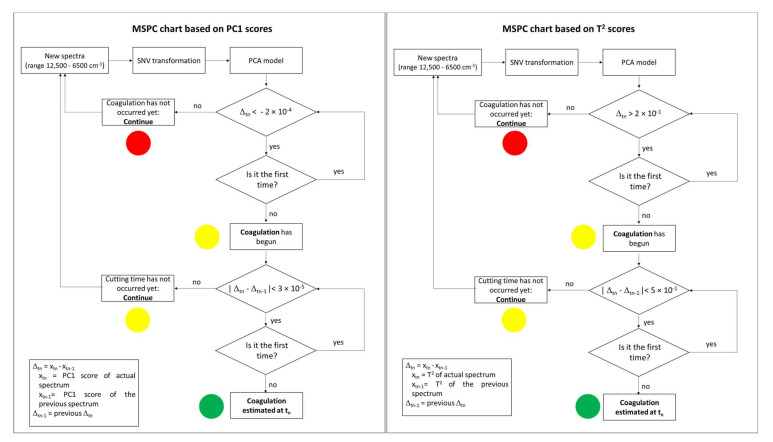
Process decision trees for real-time coagulation control from MSPC charts based on PC1 scores and T^2^.

An effective control scheme was arranged based on PC1 scores and T^2^ as described in the decision trees reported in Figure 6.

The decision trees summarize how real-time coagulation control could be performed using FT-NIR spectra collected online, to help the personnel by an automated decision-making procedure. Both decision trees estimated the beginning of the coagulation by the actual difference in PC1 scores and T^2^, whereas the end of the coagulation was defined by the absolute difference between two consecutive spectra (Δ_tn_ − Δ_tn−1_). The assessment of the two key moments was based on specific thresholds in Δ_tn_ or |Δ_tn_ − Δ_tn−1_|, defined according to the deviation of PC1 and T^2^ after the coagulation occurrence according to rheological measurements. A further control was added to manage possible noise in the recorded measures; in detail, the specific condition in the decision points should be repeated twice in a raw to confirm the coagulation start or end. The decision tree could be easily converted into a simple light signal to be interpreted by the personnel managing the coagulation process. Indeed, a red signal would appear in the control system up to the beginning of the coagulation, i.e., when Δ_tn_ overcomes two times the defined limits. At this moment, the light signal would convert into yellow up to the fulfilment of the second condition of the decision tree, i.e., when the value of |Δ_tn_ − Δ_tn−1_| is below the defined threshold for two consecutive times. At the estimated coagulation time, the light would convert into green, communicating to the personnel that the coagulation phase is over.

According to the presented decision tree schemes, the coagulation’s beginning and end occurred at specific times for the EPI40- and EPI60-replicated batches (Table 2), similarly to the rheological gelation times. On the other hand, for FB2 and FB3, neither the beginning nor the ending times were defined by any of the control charts. For FB1, only T^2^–MSPC chart detected a failure in coagulation occurrence; indeed, the predicted times were delayed with respect to the optimal time for processing. Thus, the use of T^2^ resulted in the best option for control charts, not only to assess the coagulation occurrence in normal operating conditions, but also for failure detection.

## 4. Conclusions

The present work aimed at closing the gap between the proved scientific reliability of NIRS for milk coagulation monitoring and the industrial criteria needed for applying PAT tools in a process. The comparison of FT-NIR spectral changes over time with rheological time–curing curves confirmed the capability of NIRS to monitor the desired critical quality parameter, i.e., the coagulation occurrence. The tested fast acquisition procedure (10 s for each full spectrum acquisition) fulfilled the optimal measurement conditions necessary for real-time coagulation monitoring. The testing of the developed decision trees, based on MSPC charts, with gradually incoming data (both PC1 scores and T^2^ statistics) simulating a production situation, confirmed the suitability of the proposed approach for the prediction of the coagulation times and the detection of possible failures.

In conclusion, the work provides a robust but simple system to assist skilled personnel in following milk renneting by the conversion of the proposed method into a simple traffic light signal to be visualized by the operators.

Further research could be focused on the effects of milk acidification before renneting by means of organic acids and/or starter cultures and on the use of concentrated milks obtained by membrane filtration (i.e., ultrafiltration/microfiltration).

## Figures and Tables

**Table 1:** Rheological parameters extrapolated from the elastic modulus curves obtained for each replicate of the mix ratio coagulation trials.

Trial	Rheological Estimate of Gelation Time (s)	Rheological Estimate of Cutting Time (s)
EPI40-R1	380	1270
EPI40-R2	350	1240
EPI40-R3	370	1230
EPI60-R1	330	1110
EPI60-R2	300	1020
EPI60-R3	300	1010

R1, R2 and R3 correspond to the three replicates of each mix ratio tested.

**Table 2:** Coagulation occurrence time predicted by the developed decision trees based on PC1 and T^2^ MSPC charts.

	PC1-MSPC Chart		T^2^-MSPC Chart	
Trial	Beginning (s)	End (s)	Beginning (s)	End (s)
EPI40-R1	120	420	120	420
EPI40-R2	120	480	120	480
EPI40-R3	120	420	120	420
EPI60-R1	120	360	120	360
EPI60-R2	120	360	120	360
EPI60-R3	120	360	120	360
EPI40-A	50	320	140	320
EPI40-B	70	330	130	280
EPI40-C	80	330	140	340
EPI60-A	80	220	130	290
EPI60-B	80	230	130	280
EPI60-C	60	230	130	230
FB1	70	380	210	490
FB2	-	-	-	-
FB3	-	-	-	-

R1, R2, and R3 correspond to the three replicates of each mix ratio tested with the 64-scan procedure; A, B, and C correspond to the three replicates of each mix ratio tested with the 9-scan procedure. FB1, FB2, and FB3 correspond to failure batches: in FB1 milk, heating was turned off after rennet addition; in FB2, half of the rennet amount was added; in FB3, half of the CaCl_2_ amount was added. MSPC: multivariate statistical process control.

## Data Availability

The datasets generated for this study are available on request to the corresponding author.
